# Validation of the Motivated Strategies for Learning Questionnaire among clinical clerkship students in Malaysia

**DOI:** 10.1371/journal.pone.0319763

**Published:** 2025-04-08

**Authors:** Sahar Fatima, Vinod Pallath, Wei-Han Hong

**Affiliations:** 1 Medical Education & Research Development Unit (MERDU), Faculty of Medicine, Universiti Malaya, Kuala Lumpur, Malaysia; 2 Medical Education & Assessment, Jeffrey Cheah School of Medicine and Health Sciences, Monash University, Bandar Sunway, Selangor, Malaysia; Ankara University Faculty of Medicine: Ankara Universitesi Tip Fakultesi, TÜRKIYE

## Abstract

The 81-item Motivated Strategies for Learning Questionnaire (MSLQ) is a validated tool for measuring self-regulated learning (SRL) and comprises two scales, Motivation and Learning strategies. Although its use in health professions education has been well established, validation in clinical clerkship years is scant. This study aims to investigate the structural validity and internal consistency of MSLQ in the context of Malaysian clinical clerkship students. The data from 349 clinical clerkship students, who completed the adapted MSLQ was used to test the internal consistency and hypothesised factor structure using confirmatory factor analysis for both the Motivation and Learning strategies scales. For the Motivation scale, the Cronbach’s alpha values ranged from 0.63 to 0.90 and for the Learning strategies scale from 0.61 to 0.86, indicating acceptable to high internal consistency. Cronbach’s alpha for whole MSLQ was 0.91, suggesting an excellent reliability of MSLQ in our sample. The model fit indices for the Motivation scale were comparable to the original MSLQ indicating a reasonable fit whereas the model fit indices for the Learning strategies scale did not fit well with our sample and required re-specification to attain a marginal fit. After re-specification for a better model fit, the six-factor Motivational scale and nine-factor Learning strategies scale retained their original constructs in our population sample of clinical clerkship students. Comparing our findings with the original MSLQ and previous studies, we can claim a marginal fit of our data and the theoretical model and suggest the need for further testing with a larger sample size and across different institutions. We recommend that the 75-item re-specified MSLQ-CL could be valuable for future SRL investigations among clinical clerkship students in general as well as in context of Asian medical education context.

## Introduction

Medical doctors encounter fast-paced developments in medical science. They are frequently confronted with newer guidelines, advancements in drugs and therapy concepts, and are therefore, required to develop the core competence of lifelong learning and self-regulation [1]. Self-regulation or Self-regulated learning (SRL) is the learning where learners are “metacognitively, motivationally, and behaviourally proactive in the learning process” [2]. Self-regulated learners effectively track their progress towards pre-set goals, evaluate their learning strategies, and actively engage in behaviours that optimise their learning outcomes.” [2,3].

The social cognitive perspective suggests that self-regulated learning results from the interaction between personal, behavioral, and environmental factors [4,5]. This dynamic interplay emphasises the contextual nature of SRL, where individuals adapt their strategies to specific environments, highlighting the influence of context on effective self-regulation [6,7]. The individual’s ability to effectively self-regulate can vary significantly across different environments [5]. Self-regulated learning in a clinical setting is distinct from that in an academic environment. Learning within the clinical learning environment is more opportunistic and dependent on the setting, patients, supervisors, and learner needs [8]. Despite being more autonomous in controlling their learning, clinical clerkship students cannot solely concentrate on achieving their own learning goals because they are ancillary to the provision of health care to the patients [9]. Limited time, high stakes exam pressures, patient interactions, and the dynamics of working within healthcare teams can influence how SRL manifests [10,11] and thus create a distinct context that differs significantly from other educational contexts. Hence, students who effectively manage their learning in pre-clinical, classroom environments may encounter difficulties when attempting to apply the same skills in more dynamic and demanding clinical settings [12].

Research over the past two decades has shown challenges in implementing SRL strategies in Asian medical schools [13–16]. The shared values within Asian educational culture differ significantly from West particularly in learner autonomy and student-teacher dynamics. Key differences include a strong emphasis on teacher-driven instruction in Asian pre-university education, students’ preference for traditional education, insufficient self-efficacy, over-emphasis on assessments, work overload, and insufficient organisational support for flexible curricula to promote student autonomy [13,17,18]. Prior learning experiences in exam-oriented and hierarchical educational systems may affect Asian students’ self-efficacy, intrinsic motivation, ability to set their own goals, collaboration with peers, and adapting newer approaches to learning [13,18].

The established association between SRL and enhanced academic and clinical skills performance in medical education [19] necessitates a thorough understanding of how medical students engage in self-regulated learning within specific context of clinical learning environment. Given the diverse theoretical frameworks and cultural influences on SRL [20], a culturally sensitive and psychometrically sound instrument is needed for accurately assessing SRL in this specific context. However, many researchers have expressed concerns about the suitability of SRL questionnaires in Asian contexts due to their original alignment with Western cultural and educational values [21].

The Motivated Strategies for Learning Questionnaire, MSLQ [22] is a widely utilised, validated tool for measuring SRL based on the social cognitive theory of learning [22,3]. The 81-item MSLQ [22] comprises two scales, Motivation and Learning strategies. The Motivation scale evaluate students’ goal-orientation and value beliefs as well as their confidence in their ability to achieve their targets, and their anxiety levels related to tests or exams [22]. The Learning strategies scale measure how well students apply various cognitive and metacognitive strategies, and manage various resources [22]. The scales are constructed to be modular and adaptable, catering to the requirements of the researcher or instructor [23].

The instrument manual for MSLQ states that the range of Cronbach’s alpha coefficients for internal consistency reliability was.52 to.93, indicating a satisfactory level of reliability [23]. Pintrich and colleagues [3] conducted two separate Confirmatory Factor Analyses (CFAs) on the 6-factor, 31-item Motivation scale (with a chi-square value of 3.49, GFI = .77, AGFI = .73, and RMR = .07) and the 9-factor, 50-item Learning Strategies scale (with a chi-square value of 2.26, GFI = .78, AGFI = .75, and RMR = .08). Acceptable goodness of fit was claimed pointing to a theoretically sound structure for the two MSLQ scales. The original student populations for MSLQ development and validation were primarily consisted of undergraduate college students majoring in 14 subjects in the United States [22,3]. However, the concepts of self-regulated learning and the identification of suitable measures are likely to be equally beneficial in supporting medical students [24–26]. In this study, we adapted all 81 items from the original MSLQ with minor changes suitable for the clinical clerkship context.

Although previous data on MSLQ’s reliability and validity suggest it to be a reasonably valid tool for assessing SRL in medical education [27] validity is context-specific [28], and there are compelling reasons to explore how it operates within the population of clinical clerkship students. Despite its extensive use in medical education context with pre-clinical [26,29–36], clinical [37–40], and postgraduate learners or residents [27,41], there is scarcity in research reporting MSLQ validation in clinical clerkship context. We found only 2 studies in the recent past that tried to validate MSLQ with clinical years students, that too used either a part of MSLQ or a shortened version. Dayel et al. [42] validated only Motivation scale of MSLQ in clinical years students and found satisfactory internal consistency but unsatisfactory model fit of Motivation scale for the specific context of Saudi Arabian medical students. Moreover, their sample consisted of only male students, thus raising further concerns over validation results. Fakhri et al. [38] validated a 32-item modified version of MSLQ, to measure reflection among clinical years students in Iran. Cook et al. [27] validated only Motivation scale with 120 residents and found a borderline fit with the theoretical framework, suggesting a five-factor Motivation scale without removing any items.

The emphasis on the cross-cultural and contextual differences in SRL and its measuring tools’ validation has been highlighted in numerous higher education studies [21,43,44]. The cultural nuances are also reflected in the adaptation and validation studies of MSLQ in Asian contexts. Validation of an adapted Japanese version of MSLQ was recently done with pre-clinical medical students in a Japanese medical school [45]. Japanese version (all 81 items) was adapted in context of PBL. EFA suggested a six factor, 30-item Motivation Scale and five-factor 50-item Learning strategies scale whereas CFA was not done due to smaller sample size. The authors explained the discrepancies in the learning strategies model fit in context of different educational environment and opportunities in local medical education context in Japan, as well as the content validity in terms of understanding and responding to different learning strategies questions for Japanese students. Nausheen [44] reported that the factors of Control of Learning Beliefs and Intrinsic Goal Orientation were not identified as separate constructs in Pakistani student samples. Similarly, research with Chinese students revealed diversion from original factor structure in the inter-correlation between Test Anxiety and Self-efficacy, suggesting that Chinese students, accustomed to a highly exam-oriented system, may be less affected by test anxiety compared to their American counterparts [46].

The MSLQ has been extensively adapted and utilised in the Malaysian higher education context to assess students’ levels of motivation and engagement in self-regulated learning [47]. In Malaysia, MSLQ has been used among undergraduate university students [48,49] in various fields like IT/Computers [50], Business and Finance [51,52], Teacher training [53,54], English language students [55], nursing students [56], Arabic language course [57,58], Engineering [59], Science and social sciences [60]. It has been used among pre-clinical medical students in a Malaysian medical school, mentioning the adaptation to medical context with acceptable reliability values [30], however this study did not report any structural validation. The modified and shortened versions of MSLQ have been validated in science and social sciences university students [47], and Arabic course students [58] in Malaysian universities. Thus it can be concluded that MSLQ is acknowledged as a reliable and valid tool for examining SRL among university students in Malaysia [47]. However, to our best knowledge MSLQ is not validated in medical education context, and more specifically in clinical clerkship years in Malaysia.

These data indicate potential usefulness of the MSLQ in higher and medical education, as well as the critical role of specific contexts, learning environment, and socio-cultural factors on the validity of its results. Therefore, it is reasonable to question whether MSLQ scores will yield similar results in a complex and dynamic context of clinical clerkship. As students advance into clinical years, they undergo significant personal and professional growth. This transformative period, characterised by increased clinical exposure, can significantly impact their metacognitive abilities and self-efficacy. Consequently, their self-regulatory strategies may evolve, potentially leading to distinct factor structures in their SRL measurements. Moreover, social relationships within clinical settings also influence SRL. The degree of support from peers and mentors can either facilitate or hinder self-regulation processes [11,61]. The complex interplay of contextual factors, developmental changes, goal-setting dynamics, and social interactions within clinical environments indicates the necessity for additional validation before utilising the MSLQ in clinical clerkship context. Moreover, the validity of test scores can be threatened by social and cultural issues [28], it could be argued that the functionality of MSLQ may vary within the population of Malaysian clinical clerkship students. An SRL measuring instrument should be valid and reliable to be used in various populations or context, and at the same time should be sensitive to differences in ability and comprehension levels of respondents. The measurements from such an instrument can provide insights to educators to assess learners’ SRL and design and implement strategic SRL training and focused interventions to develop effective SRL skills in learners. Therefore, this study not only fills the gap in under studied MSLQ validation research in the clinical clerkship context but also addresses the cultural adaptation and applicability of the instrument in Asian or more specifically Malaysian medical education context. This study will contribute to the knowledge of the generalisability of MSLQ to clinical years student population and the utility of the instrument in understanding SRL in similar content and contexts. The study thus aims to investigate the structural validity and reliability of Motivation and Learning strategies scales of 81-item MSLQ in context of clinical clerkship students in a Malaysian medical school. The objectives of the present study are:

1. To evaluate how well the theoretical model of MSLQ aligns with empirical data through Confirmatory Factor Analysis (CFA).
2. To describe the internal consistency of MSLQ scores in clinical clerkship students.

Our study adds significantly to the literature by assessing validity of MSLQ in a previously under-studied population. Notably, we found only one study that employed CFA to assess the validity of the original MSLQ (only Motivation scale) among clinical clerkship students [42]. Furthermore, there have been no such validity assessments conducted in Malaysia.

## Methods

### Setting and sample

This study was conducted in a public university in Malaysia between November 2022 and June 2023. Formal approval was obtained from the University Malaya Research Ethics Committee (UMREC) with Reference Number: UM.TNC2/UMREC – 1001. The five-year medical programme at the Faculty of Medicine, Universiti Malaya, Kuala Lumpur, consists of Year 1 and Year 2 pre-clinical years (referred to as Stage 1 and Stage 2) followed by Year 3, Year 4, and Year 5 (referred to as Stages 3.1, 3.2 and 3.3 respectively) where clinical clerkship at the teaching hospital takes place. All 476 medical students in the clinical clerkship years, i.e., Stage 3.1, 3.2, and 3.3 were invited to participate, out of which 349 students completed the survey.

### Adaptation of MSLQ

The 81-item original MSLQ [23] was adapted in accordance with the context of clinical clerkship. The 81 items of the MSLQ are scored on a 7-point Likert scale (1 being strongly disagree and 7 being strongly agree). It comprises two scales: i.e., Motivation and Learning strategies [23]. The Motivation scale is divided into six sub-scales namely Intrinsic Goal Orientation, Extrinsic Goal Orientation, Control Of Learning Beliefs, Self-Efficacy, and Test Anxiety [3]. The Learning strategies scale is divided into nine sub-scales namely Rehearsal, Elaboration, Organisation, Critical Thinking, Metacognitive Self-Regulation, Time/Study Environment Management, Effort Regulation, Peer Learning And Help-Seeking [3]. Some amendments were made in the wordings of the original questionnaire to suit the context of learning in clinical clerkship context (complete amendments are provided as supporting information S1 Table). The refinement complied with the administration instructions of the authors [23] suggesting it can be used to fit the needs of the researcher in a given context. The content was discussed through several rounds of discourse among the members of our research team. Some examples of the phrases’ refinements in the questionnaire are shown in Table 1.

**Table 1:** Examples of word/phrase amendments in MSLQ during initial adaptation and after content validity.

Original phrase/word	Initial amendments	Amendments after content validity
course	clinical posting	
Test/exam	assessment	
course content	content of clinical posting	
lecturer	tutor	
material	content	
When I take an assessment		When I face an assessment
When studying for this class, I read my class notes and the course readings over and over again.	When studying for this clinical posting, I read my notes and the study material over and over again.	When studying for this clinical posting I do deliberate practice of my tasks repeatedly.

### Content validity of adapted MSLQ

Content validity is a fundamental prerequisite and minimum requirement for instrument validation, ensuring the appropriateness and representativeness of its content and development process [62]. Therefore, we started the process of adapted MSLQ validation by establishing its content validity. A panel of experts were approached to review instrument components and rank them according to how closely they relate to and represent the content domain [62]. We selected the experts panel on the basis of their clinical teaching and medical education expertise, who not only were familiar and experienced in teaching medical students in clinical years, but also had sound knowledge of conceptualisation, theoretical frameworks and learning theories of self-regulation used in the context of the present study [63,64]. Currently, there is no consensus on any fixed number of experts to review an instrument but most literature suggests using 5-10 experts in the content validation process [62]. We invited ten experts via email and eight agreed to review the questionnaire. The experts were provided the content validity form with clear instructions of the scale and scoring along with the operational definitions of the main constructs to facilitate the rating of items in context of present study. Content validity for each item (I-CVI) and entire scale (S-CVI) were calculated. I-CVI (item-level content validity index) is the proportion of content experts giving item a relevance rating of 3 or 4, calculated by number of experts who rated the item as 3 or 4/ total number of experts. The recommended values for I-CVI is ≥  0.78. S-CVI (scale-level content validity index) is the average of the I-CVI scores for all items on the scale or the average of proportion relevance judged by all experts, calculated by sum of I-CVI scores)/(number of items). The recommended values for S-CVI ≥  0.9 [62], though some literature has reported S-CVI ≥  0.8 as acceptable [63]. In the present study most of the items had an I-CVI of ≥  0.78 and the S-CVI for the whole questionnaire was 0.84, rendering it to be acceptable. The items that had I-CVI of <  0.78 were reviewed and it was decided to revise a number of items and retain all items for next phases of validation. Based on the content validity and reviewers’ suggestions, changes were made in the adapted MSLQ as shown in Table 1 (complete amendments are provided as supporting information S1 Table). The items were also evaluated for their linguistic and contextual appropriateness for use in the Malaysian context. The profile of panel of experts and content validity calculations for I-CVI and S-CVI are provided as supplementary material S1 Appendix.

### Pilot study

A recruitment email with a request to fill the attached questionnaire on Google Form was sent to 45 medical students who were informed about the purpose of the pre-testing, and that their participation is entirely voluntary and will be kept anonymous. The students were from Years 3, 4, and 5 of clinical clerkship, of the same age group as present study sample, had undergraduate entry and completed 2 years of pre-clinical MBBS curriculum at a private medical school in Malaysia. Out of 45 students, 32 students responded. Participants were asked to give a feedback in the feedback section of Google Form questionnaire regarding the clarity, relevance and further improvement in items. All items were answered by respondents who did not report any problems on the survey. The Cronbach alpha reliability for the adapted MSLQ was 0.9.

### Data collection

Students were given information on the study and that their responses would help researchers better understand the motivation and strategies during the learning process among clinical clerkship students. The questionnaire was administered in Google Forms format. Written informed consent from the participants was obtained at the start of the questionnaire. Data was collected towards the end of each academic year when they have completed most of the clinical postings of the respective years. Recruitment of participants continued from 18^th^ October 2022 till 16^th^ May 2023. A total of 349 students completed the questionnaire, yielding a response rate of 73%, including 125 Year 3 or Stage 3.1 (35.8%), 143 Year 4 or Stage 3.2 (41.0%) and 81 Year 5 or Stage 3.3 (23.2%) clinical clerkship students. The participants’ ages ranged from 21 to 26 years at the time of data collection. The gender distribution was slightly dominated by the female population with 58.7% female and 39.5% male, whereas 1.7% of participants did not provide gender information. All 349 responses were included in the data analysis.

### Data Analysis

The MSLQ’s factor structure was subjected to confirmatory factor analysis (CFA) in order to look into its psychometric qualities. The factor structure delineates the connections between the observed variables, which are the measured items, and the latent factor or construct being estimated [65]. In the context of research participants, Confirmatory Factor Analysis (CFA) serves as a means to verify the construct validity of the expected factor structure [66]

The following fit indices are reported in our CFA because they are the most widely reported indices in the literature [21] and should be included in any CFA study to assess the model fit between the hypothesized model and observed data [67,68]: (a) CMIN/DF, with a suggested threshold value < 3 for a good model fit [69–71]; (b) root-mean-square error of approximation (RMSEA), with a suggested threshold value < .08 [72,73]; (c) standardized root mean squared residuals (SRMR), with a recommended value ≤ .10 [72]; and (d) comparative fit index (CFI), with a suggested acceptable value > .90 although values between 0.8-0.89 can be termed marginal fit [65,66]. We reported the values of the Goodness of Fit Index (GFI), and the Adjusted Goodness of Fit Index (AGFI) to compare our results with the original validation of MSLQ by its developers [3]. However, reporting GFI and AGFI was not recommended by Hu and Bentler [74] as absolute fit indices due to their insensitivity to errors in model specification.

CFAs were conducted using IBM SPSS Amos 26 [75]. Maximum likelihood was employed to generate parameter estimates, and goodness-of-fit tests were conducted with freely allowed correlations between factors (depicted in supporting information data in S1 Text). The negative worded items in the Learning strategies scale (items 33, 37, 40, 52, 57, 60, 77 and 80) were reverse coded. Descriptive statistics and internal consistency reliability estimates were computed for both the default and modified MSLQ scales using IBM SPSS Statistics (Version 26).

## Results

### Model Comparisons

Similar to Pintrich and colleagues’ validation of MSLQ [3] two separate Confirmatory Factor Analyses (CFAs) were carried out for the Motivation and Learning strategies scales of the MSLQ. The 31 Motivation items were subjected to the first maximum likelihood estimation to assess the fit of a six-factor model with correlated factors to the data. The 50 items related to Learning strategies were subjected to a second round of maximum likelihood estimation to evaluate the fit of a correlated nine-factor model to the data. The model fit indices for each MSLQ section to the clinical clerkship sample data along with the model fit indices of the original MSLQ [3] and acceptable values, are shown in Table 2.

**Table 2:** CFA fit statistics for Motivated Strategies for Learning Questionnaire (MSLQ).

Fit Indices	Recommended values	Original MSLQ (Pintrich 1993)		Default Model		Re-specified Model	
		**MOT**	**LS**	**MOT**	**LS**	**MOT**	**LS**
Chi-sq/df	< 3	3.49	2.26	3.14	2.98	2.94	2.80
GFI/AGFI	> 0.9	0.77/0.73	.78/.75	0.78/0.74	0.67/0.63	0.80/0.76	0.74/0.70
CFI	> 0.9			0.82	0.710	0.83	0.78
SRMR	< 0.10			0.08	0.09	0.08	0.07
RMSEA	< 0.08			0.07	0.07	0.07	0.07

Results for the Motivation scale were comparable to those originally described by Pintrich as “the six correlated latent factors model appears to be the best fitting representation of the input data” [3] as shown in Table 2. The chi-square/df was 3.14, which is just above the recommended value of < 3. GFI/AGFI was 0.78/0.74, CFI 0.82, i.e., well below the preferred value of >  0.9 but closer to 0.8 for a marginal fit, RMSEA was 0.078 (with approximately 90 percent confidence, the population RMSEA is between.074 and.083) and SRMR 0.08; well within the acceptable range. The factor loading estimates were >  0.32, the cut-off value to retain the items. Although the CFI value does not comply with the ideal model fit values, we can say that it was closer to a marginal fit, comparable to the original MSLQ and might be considered for re-specification for an improved fit.

For the Learning strategies scale the chi-square/df was 2.98, which is acceptable. GFI/AGFI was 0.67/0.63 quite lower than those of original MSLQ values, CFI 0.71 which is not acceptable or close to marginal fit, RMSEA was 0.076 (with approximately 90 percent confidence, the population RMSEA is between.073 and.078) and SRMR 0.09; well within the acceptable range, as shown in Table 2. The factor loadings of a few items were below the acceptable value of 0.32. Despite the acceptable values of chi-square/df, SRMR and RMSEA, the model does not fit the data well and requires re-specification.

### Re-specification of MSLQ

#### Motivation Scale.

**Fig 1:**
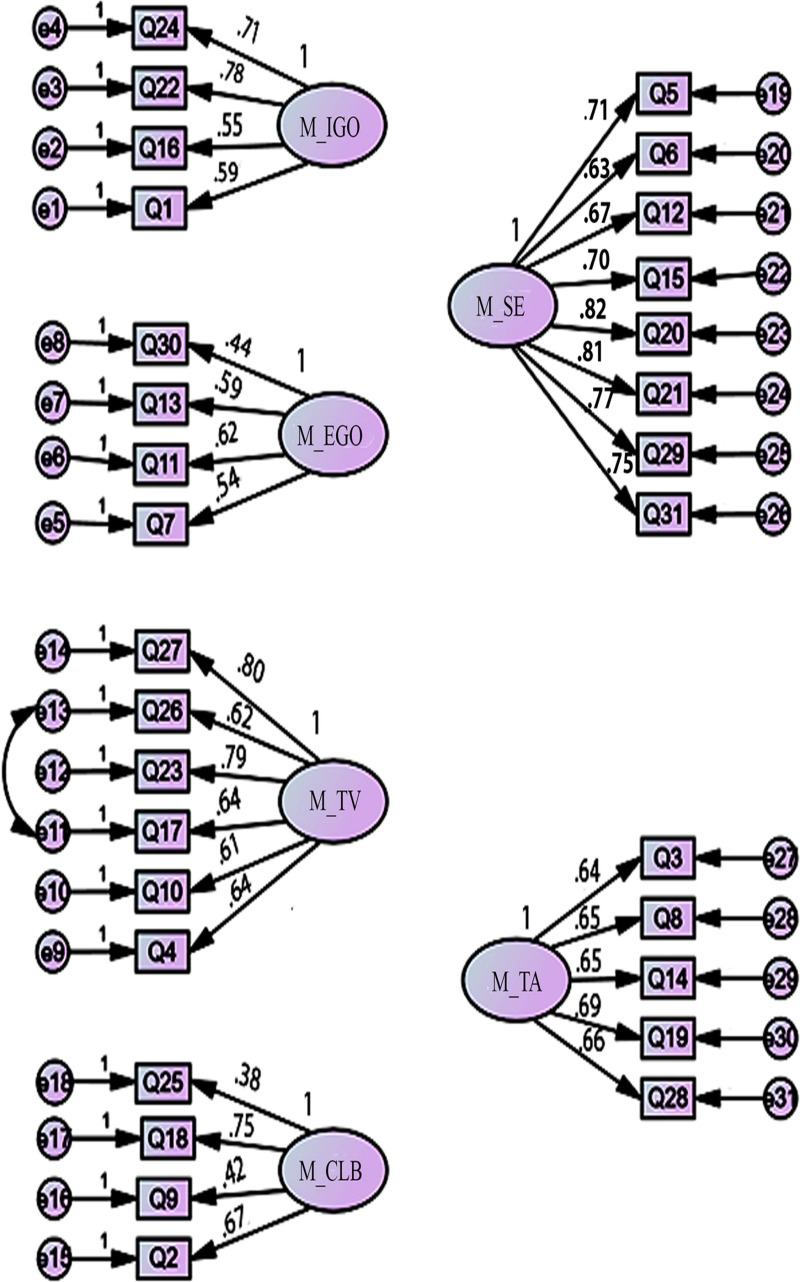
Path diagram of six-factor model for Motivation scale showing standardised factor loadings.

We examined a first-order latent six-factor model of the motivation scale of MSLQ, allowing the six factors to correlate. The factor loadings of all 31 items were ≥  0.35 (shown in Fig 1) and were retained. The error variances of items 17 and 26 were correlated. According to Byrne et al [76], error variances can be correlated if they belong to the same construct, have some redundancy/overlap in their content so the respondents may respond to both items quite similarly or the same items error variances were correlated in previous studies. Items 17 and 26 belong to the same construct of Task value. Their mean scores from our sample data are 5.39 (item 17) and 5.40 (item 26), indicating our participants responded to both items in a similar way. The error variances of the same items were also correlated in previous studies to improve model fit [42,77].

IGO =  Intrinsic Goal Orientation; EGO =  Extrinsic Goal Orientation; TV =  Task Value; CLB =  Control of Learning Beliefs; SE =  Self-Efficacy; TA =  Test Anxiety (All factors were allowed to corelate)

All the Motivation sub-scales, except for Test Anxiety, were positively correlated with each other as shown in Table 3 (upper right). Given the limitations of Cronbach’s alpha for comparing subscales with varying item numbers, as suggested by the Spearman-Brown prophecy [78], we also report median inter-item correlations. This provides a more robust and comparable indicator of internal consistency across subscales with different lengths.

**Table 3:** Means, reliabilities, and between-domain correlations for Motivation scale of Motivated Strategies for Learning Questionnaire (MSLQ).

	Mean (SD) range	IGO	EGO	TV	CLB	SE	TA
IGO	5.17 (.90) 2.25-7.00	**0.762** **(0.447)**	0.743	0.880	0.775	0.730	0.038
EGO	5.18 (.98) 2.50-7.00	.475	**0.635** **(0.306)**	0.781	0.765	0.566	0.306
TV	5.66 (.81) 2.00-7.00	.675	.518	**0.844** **(0.477)**	0.943	0.642	0.126
CLB	5.43 (.89) 2.25-7.00	.450	.447	.592	**0.645** **(0.301)**	0.546	0.250
SE	4.76 (.93) 1.00-7.00	.623	.408	.598	.258	**0.901** **(0.534)**	-0.307
TA	5.00(1.14) 1.40-7.00	.014	.225	.073	.297	-.261	**0.792** **(0.433)**

Items along the diagonal (bold) are Cronbach’s alpha with median inter-item correlation in parenthesis. Values in the lower left represent Pearson’s correlation coefficients. Values in the upper right (shaded) represent correlation estimates from confirmatory factor analysis

n = 349 for all analyses.

SD =  standard deviation; IGO =  intrinsic goal orientation; EGO =  extrinsic goal orientation; TV =  task-value; CLB =  control of learning beliefs; SE =  self-efficacy; TA =  test anxiety.

#### Learning strategies scale.

**Fig 2:**
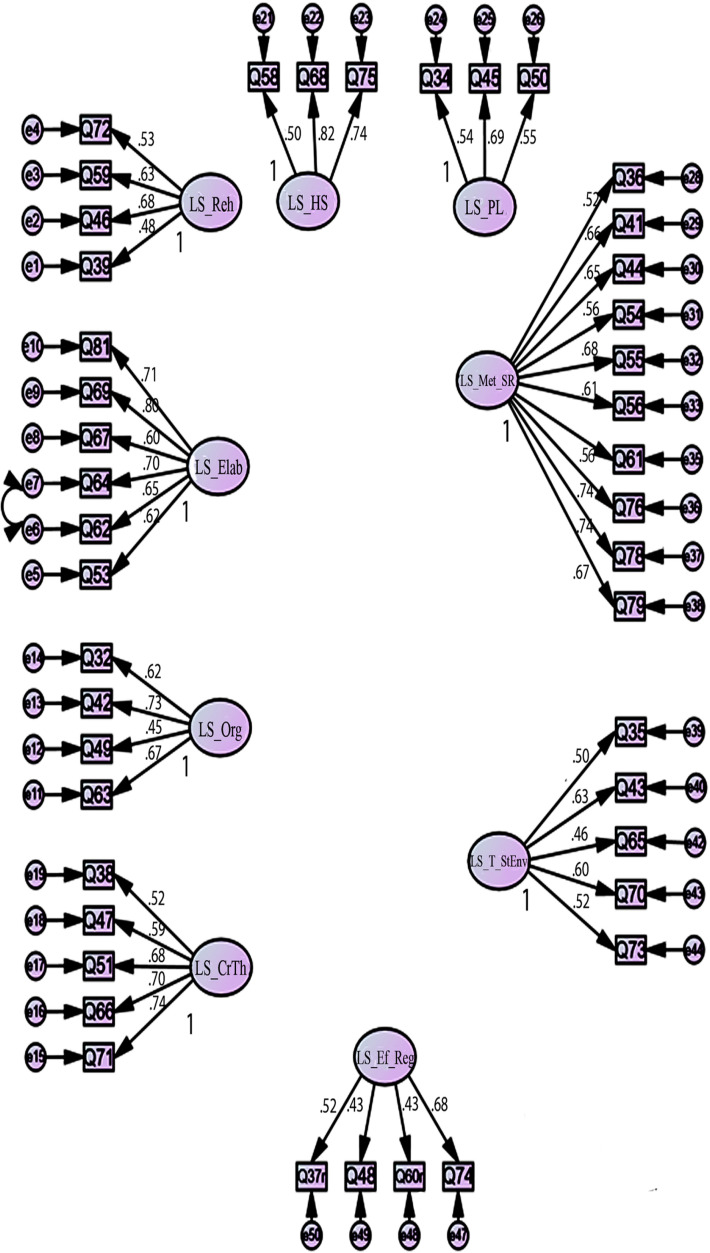
Path diagram of nine-factor model for Learning strategies scale showing standardised factor loadings. Reh =  Rehearsal; Elab =  Elaboration; Org =  Organisation; Crth =  Critical Thinking; Met_SR =  Metacognitive Self-Regulation; T_StEnv =  Time And Study Environment; Ef_Reg =  Effort Regulation; PL =  Peer Learning; HS =  Help Seeking (All factors were allowed to corelate).

We examined a first-order latent nine-factor model of the learning strategies scale of MSLQ, allowing the nine factors to correlate. It was desirable to keep the original nine factor structure of the learning strategies scale with a minimum of three items per factor to avoid identification problems. The factor loadings of six items (items 33,57 from Metacognitive Self-regulation, items 52,77,80 from Time and Study Environment, item 40 from Help Seeking) were less than the set threshold of 0.32 [21,79]. One-by-one, items were removed to observe alterations in the factor structure solution and construct the optimal fit and most concise model for the data, while maintaining the original factor structure. The factor loadings of remaining items were >  0.40 as shown in Fig 2. The error variances of items 62 and 64 were correlated. Items 62 and 64 belong to the same construct of elaboration. Their mean scores from our sample data are 5.31 (item 62) and 5.47 (item 64) indicating participants responded to both items in a nearly similar way. The error variances of the same items were also correlated in previous studies to improve model fit [77].

The re-specification process resulted in a 44-item model with improved model fit indices as indicated in Table 2. As expected, all Learning strategies subscales were positively correlated to one another as shown in **Table 4** (upper right).

**Table 4:** Means, reliabilities, and between-domain correlations for Learning Strategies scale of Motivated Strategies for Learning Questionnaire (MSLQ).

	Means(SD) range	Reh	Elab	Org	CrTh	Met_SR	T_StEnv	Ef_Reg	PL	HS
Reh	4.93(0.97) 1.00-7.00	**0.673** **(0.348)**	0.862	0.906	0.816	0.956	0.891	0.735	0.931	0.644
Elab	5.30(0.89) 2.00-7.00	0.612	**0.823** **(0.491)**	0.904	0.735	0.934	0.895	0.735	0.790	0.693
Org	5.01(0.98) 2.00-7.00	0.611	0.688	**0.700** **(0.403)**	0.679	0.838	0.816	0.735	0.719	0.474
CrTh	4.72(0.97) 1.2-7.00	0.580	0.632	0.534	**0.784** **(0.405)**	0.872	0.731	0.510	0.770	0.524
Met_SR	0.48(0.92) 1.00-7.00	0.709	0.824	0.679	0.750	**0.866** **(0.414)**	0.940	0.749	0.825	0.638
T_StEnv	5.3(0.86) 2.2-7.00	0.566	0.677	0.534	0.541	0.713	**0.674** **(0.284)**	1.018	0.699	0.620
Ef_Reg	4.68(1.05) 1.00-7.00	0.371	0.501	0.421	0.292	0.482	0.599	**0.611** **(0.280)**	0.623	0.457
PL	4.81(1.05) 1.00-7.00	0.605	0.561	0.484	0.574	0.619	0.442	0.337	**0.625** **(0.392)**	0.872
HS	5.31(1.10) 1.00-7.00	0.478	0.564	0.367	0.460	0.564	0.509	0.337	0.594	**0.701** **(0.336)**

Items along the diagonal (bold) are Cronbach’s alpha with median inter-item correlation in parenthesis. Values in the lower left represent Pearson’s correlation coefficients. Values in the upper right (shaded) represent correlation estimates from confirmatory factor analysis.

n =  349 for all analyses.

SD =  standard deviation; Reh =  Rehearsal; Elab =  Elaboration; Org =  Organisation; Crth =  Critical Thinking; Met_SR =  Metacognitive Self-Regulation; T_StEnv =  Time And Study Environment; Ef_Reg =  Effort Regulation; PL =  Peer Learning; HS =  Help Seeking.

### Internal consistency of MSLQ

The internal consistency was examined for both the default and re-specified models. The standard deviations of all items were more than 0. The inter-item correlation between individual items within the sub-constructs of both the Motivation and Learning strategies scales was deemed satisfactory, with most items displaying strong correlations with others, barring a few exceptions. The inter-item correlations of majority of the items were seen to be within.30 to.70 and can be considered acceptable besides having at least 50% of the retained items with total scores in the range of.30 and.70 [80,81]. The median inter-item correlations of each construct of both the Motivation and Learning strategies scales are shown in Tables 3 and 4 respectively. The corrected item-total correlations for both Motivation and Learning strategies constructs were well above 0.2, the recommended cut-off value of CITC to retain an item [82,83]. For the Motivation scale, the Cronbach’s alpha values ranged from 0.63 to 0.90 and for the Learning strategies scale from 0.61 to 0.86, indicating acceptable to high internal consistency. The Cronbach’s alpha for whole MSLQ was 0.91, suggesting an excellent reliability of MSLQ in our sample [82]. Since Cronbach’s alpha assumes uni-dimensionality and tau-equivalence, we also reported Mcdonald’s Omega [84] as an alternative reliability index using the item factor loadings and uniqueness from a factor analysis [85]. The Cronbach’s alpha for original MSLQ, default model and revised model for each sub-scale along with Omega values for re-specified model are reported in Table 5.

**Table 5:** Reliability analysis of MSLQ for the present study as compared to the original MSLQ.

Sub-scales	Items	Alpha (original MSLQ)	Alpha (Default model)	Alpha (Re-specified model)	Omega (Re-specified model)
**MOTIVATION**					
IGO	1,16,22,24	0.74	0.76	0.76	0.76
EGO	7,11,13,30	0.62	0.63	0.63	0.65
TV	4,10,17,23,26,27	0.90	0.84	0.84	0.84
CLB	2,9,18,25	0.68	0.64	0.64	0.60
SE	5,6,12,15,20,21,29,31	0.93	0.90	0.90	0.90
TA	3,8,14,19,28	0.80	0.79	0.79	0.79
**LEARNING STRATEGIES**					
Reh	39,46,59,72	0.69	0.67	0.67	0.67
Elab	53,62,64,67,69,81	0.75	0.82	0.82	0.82
Org	32,42,49,63	0.64	0.70	0.70	0.69
CrTh	38,47,51,66,71	0.80	0.78	0.78	0.78
Met_SR	33,36,41,44,54,55,56,57,61,76,78,79	0.79	0.82	0.86 (removed 33r,57r)	0.86
T_StEnv	35,43,52,65,70,73,77,80	0.76	0.65	0.67 (removed 52,77r,80r)	0.66
Ef_Reg	37,48,60,74	0.69	0.61	0.61	0.59
PL	34,45,50	0.76	0.62	0.62	0.62
HS	40,58,68,75	0.52	0.54	0.70 (removed 40r)	0.74
MSLQ (range)	1-81	0.52-0.93	0.54-0.90	0.61-0.90	0.59-0.90

IGO: Intrinsic Goal Orientation; EGO: Extrinsic Goal Orientation; TV: Task Value; CLB: Control of Learning Beliefs; SE: Self-efficacy; TA: Test Anxiety

Reh =  rehearsal; Elab =  elaboration; Org =  organization; CrTh =  critical thinking; Met_SR =  metacognitive self-regulation; T_StEnv =  time and study environment; Ef_Reg =  effort regulation; PL =  peer learning; HS =  help seeking

## Discussion

This study examined the validity of MSLQ scores for assessing medical students’ self-regulated learning in clinical clerkship years. The CFA results for the Motivation scale were comparable to the original MSLQ validation [3] and only slight re-specification resulted in a better fit, which we can claim to be a reasonable model fit for our sample. At the same time, CFA results for the Learning strategies scale were not in acceptable ranges and posed psychometric issues. This is in accordance with the previous studies where the Motivation scale came out to be a better fit than Learning strategies scale [43,45]. This might be due to the concise nature of the Motivation scale that focuses on planning of goal setting at an individual level compared to more complicated descriptions of the Learning strategies scale that refer to applying strategies, monitoring performance and reflecting on the learning process [86]. In addition, cultural influences may have an impact on how learners behave in relation to the scales of profound learning (e.g., metacognitive self-regulation) and interpersonal learning (e.g., peer learning and help seeking). Our CFA results suggested a need for re-specification of the original 81-item MSLQ for our sample. This result is consistent with earlier MSLQ adaptation and validation research that determined cross-cultural modification and adaptation were required to address the applicability of SRL models from western contexts to eastern contexts [43,45,47,87,88].

The model fit indices for the Motivation scale for the default model were comparable with those of the original MSLQ [3]. The covariance of error terms for items 17 and 26 were correlated which resulted in a better model fit. This may reflect relationships between items due to similarities in the wording or content of the two items. Both items 17 and 26 loaded significantly on task value, indicating that these items shared variance with this factor. However, the sentences that make up the stems for item 17 (I am very interested in the content area of this clinical posting), and item 26 (I like the subject matter of this clinical posting) also share similar ideas of liking the content of a clinical posting and may reflect our sample’s tendency to respond similarly to both items. The error variances of the same items were also correlated in previous studies when trying to improve the model fit of the motivation scale [42,77]. The re-specified model showed improved model fit indices as compared to the default model and original model of Pintrich [3]. Although the model did not meet the ideal fit criterion of CFI >  0.9, but it can be rendered as a marginally fit CFI value, with other indices being in the acceptable range, supported by robust internal consistency, we can claim that the six-factor motivation model fits reasonably well in our data.

The model fit indices for the Learning strategies scale of the default model did not fit well with our sample. Therefore, we tried some re-specification to improve model fit by removing 6 items with low factor loadings. Similar items had posed psychometric and/or internal consistency problems in previous studies and were removed by the researchers to improve model fit [30,43,47,65,89]. The removal of items not only reflected model modification suggested by CFA (low factor loadings) but the items were not clear enough to be comprehended by our population. For example item 40 from help-seeking “Even if I have trouble learning the content in this clinical posting, I try to do the work on my own, without help from anyone” might be mistaken as positive SRL aspect of completing the tasks relying on own capabilities rather than negative SRL aspect of not seeking appropriate help, resulting in very low factor loading for help-seeking construct. In addition few items were not congruent to present study’s theoretical underpinning in context of clinical clerkships. For example, item 52 from time and study environment “I find it hard to stick to a study schedule during clinical posting” does not align with SRL adaptation of medical students in a more dynamic and fluid clinical learning environment where it’s important to manage time and space according to given situations, rather than following a fixed schedule or timetable. Similarly, two more items from Time and Study Environment, item 77r: “I often find that I don’t spend very much time on this clinical posting because of other activities”, and item 80r: “I rarely find time to review my notes or study material in this clinical posting before an assessment” primarily focus on external factors such as time constraints during clinical clerkship rather than students’ perceptions of control and adaptation over their study time within the context of clinical clerkship. Two items from Metacognitive Self-regulation, item 33r: “During clinical posting time I often miss important points because I’m thinking of other things, and item 57r: “I often find that I have been reading for this clinical posting but don’t know what it was all about.” may reflect external distractions and lack of concentration rather than perceived internal metacognitive processes which emphasize self-awareness and regulation of one’s own thinking, not necessarily the impact of external distractions. The covariance of error terms for items 62 and 64 from elaboration scale were correlated. Items 62 (I try to relate ideas in this clinical posting to those in other clinical postings whenever possible) and 64 (When reading for this clinical posting, I try to relate the content to what I already know) share somewhat similar ideas and words of relating to previous knowledge and may reflect our sample’s tendency to respond similarly to both items. The error variances of the same items were also correlated in a previous study [77]. The re-specified model showed improved model fit indices and reliability as compared to the default model and can be compared to those of the original model of Pintrich [3]. Although the model did not meet the ideal fit criterion of CFI >  0.9, but it is closer to a marginally fit CFI value, and with other indices being in the acceptable range, supported by robust internal consistency, we can claim that the nine-factor learning strategies model marginally fits in our data.

All factors in Motivation and Learning strategies were positively correlated as expected, except for Test Anxiety which was negatively or weakly positively correlated with other motivational factors. Similarly all factors in Learning strategies scale were positively correlated consistent with the original MSLQ [3] and previous studies [43,77,87]. Some of the factors in both Motivation and Learning strategies scales were very strongly correlated ( > 0.85) as to suggest that the same construct is being assessed. However, all factors distinctly measure separate theoretical constructs that are closely related to each other. For example high correlations between time and study environment and effort regulation, or between peer learning and help seeking in the present study were also reported in previous studies [90]. Both of these pairs are categorised under resource management strategies in MSLQ. They are closely linked to each other but reflect distinct measures of SRL. Time and Study Environment emphasises deliberate efforts in planning time and structuring physical environment to complete the task, whereas effort regulation encompasses strategies to keep focus on task completion in face of distractions. High correlations may also be considered under how students understood the items. Student might consider them almost similar strategies and could have answered the same way. A rewriting of items in such constructs for better comprehension is warranted. We did not intend to merge or remove any construct as we wanted to retain the original factor structure that comprehends the theoretical underpinning of our study.

A reliability generalisation meta-analysis of MSLQ reported disparities between estimates of mean reliability from their study and originally published MSLQ that might have resulted due to moderator variables like study population, context, and original item wording [91]. The variability in reliability estimates observed for MSLQ over years highlights the importance of considering the specific context and population when interpreting reliability estimates. We should not simply assume that the reliability of the MSLQ will be consistent, as it may be significantly influenced by factors such as the characteristics of the sample and the research setting.

Finally, our results are consistent with previous cross-cultural adaptation research that eliminated some items because of low factor loadings [21,43,44,47,89]. Further, all six items that were removed from the Learning strategies scale were reverse coded, which aligns with previous cross-cultural MSLQ adaptation studies that removed four to seven reverse coded items [21,43,47,92]. It is a common practice to include negatively worded items in a questionnaire. This is done, in part, to disrupt response patterns and thus maintain active response engagement by the respondent [93]. Conventionally, the inclusion of negative statements intends to prevent response bias that could affect the research findings’ validity [94]. However, as the positive and negative statements are being included together in questionnaires, it seems that the inclusion of negative statements is producing more response error [95,96]. In our study, six out of eight negative worded items in the learning strategies scale did not load well on their intended construct in our sample. Previous research shows that recoding features using negatively worded items can result in biased outcomes when estimating procedures. This is because the measurement error in these features does not effectively capture the intended information due to the nature and orientation of the items they are based on [97]. The psychometric problems with negative worded items in our sample may also be related to verbal ability [93,98], a concern that may be relevant to the Malaysian population sample where they had completed the questionnaire in English, which is not their native language. It has also been uncovered in previous studies that Malaysian population samples do not respond well to negative worded items [47,96,99,100].

## Limitations

This study on the contextual adaptation and validation of MSLQ has a few limitations. This study was conducted in one public sector university, and therefore, the results may not be generalisable to other public or private universities. While the sample size was adequate for reliable factor analysis (with over ten observations per scale item for the Motivation scale and approximately seven observations per scale item for the Learning strategies scale), it remained relatively small for Learning strategies scale, which could potentially lead to some instability in the results. In addition, this study was conducted in the context of clinical clerkships, and other student populations, like pre-clinical or postgraduate students, might respond differently. Furthermore, the construct validity of MSLQ in clinical clerkships can be refined by looking into predictive validity utilising students’ academic scores.

## Conclusion

To the best of our knowledge this is the first study that validated the original 81-item MSLQ exclusively in clinical clerkship context, resulting in a 75-item MSLQ-Cl (provided as supporting information S2 Appendix), retaining the six-factor Motivational scale and nine-factor Learning strategies scale in our sample population of clinical clerkship students. Although the CFI values were only close to marginally fit, the other CFA indices were within acceptable ranges of good model fit. The factor loadings and parameter estimates were in acceptable ranges and mostly in the expected direction. Moreover, the validity of adapted MSLQ was supported by content validity, response process through pilot study, robust internal consistency, and theoretical alignment with socio-cognitive aspects of self-regulation. Comparing our findings with the original MSLQ and previous studies, we can claim a marginal fit of our data and the theoretical model. We agree with Crede et al. [90] when they suggested in their meta-analysis that MSLQ is still considered a valuable tool in research despite its psychometric problems or probable redundancy, as it captures the most important constructs that are critical to SRL. While lack of factorial invariance across cultures may indicate that the scale functions differently in different contexts, it does not necessarily invalidate the scale [101]. Instead, it necessitates a deeper investigation into how specific items function across different contexts. We suggest that by analysing differential item functioning in terms of relative versus absolute measurement, we can gain a better understanding of the cultural and contextual nuances of the scale and interpret its findings more appropriately.

We recommend that the 75-item MSLQ-Cl could be valuable for future SRL investigations among clinical clerkship students in general as well as in context of Asian, or more specifically, Malaysian medical education context. Future research should ensure further testing of MSLQ that include predictive validity testing to address concerns about unique predictive value of some highly correlated sub-scales. For Asian students, particularly Malaysian students, re-writing or omission of negatively worded items is suggested.

## Supporting Information

S1 Table MSLQ initial adaptation and amendments after content validation.
(PDF)

S1 Text Amos output text with default settings and parameter estimates.
(PDF)

S1 Appendix MSLQ content validation.
(XLSX)

S2 Appendix MSLQ-CL.
(PDF)

S1 Dataset Raw data generated from the study.
(XLSX)
