# Supplementary material for: Validation of the Motivated Strategies for Learning Questionnaire among clinical clerkship students in Malaysia
Source: PLoS One. 2025 Apr 8;20(4):e0319763. doi: 10.1371/journal.pone.0319763 (PMC11978056; doi:10.1371/journal.pone.0319763)
Supplement: S1 Table — (PDF) [file pone.0319763.s001.pdf]

## MSLQ initial adaptation and amendments after content validation

| Item | Original Item | Initial adaptation | Amendment after Content validation |
|---|---|---|---|
| 1. | In a class like this, I prefer study material that really challenges me so I can learn new things. | In a clinical posting, I prefer study material that really challenges me so I can learn new things. | |
| 2. | If I study in appropriate ways, then I will be able to learn the material in this course. | If I study in appropriate ways, then I will be able to learn the content in this clinical posting. | |
| 3. | When I take a test I think about how poorly I am doing compared to other students. | When I take an assessment in this clinical posting, I think about how poorly I am doing compared to other students. | When I face an assessment in this clinical posting, I think about how poorly I am doing compared to other students. |
| 4. | I think I will be able to use what I learn in this course in other courses. | I think I will be able to use what I learn<br>in this clinical posting in other clinical posting. | |
| 5. | I believe I will receive an excellent grade in this class. | I believe I will receive an excellent grade for<br>this clinical posting. | |
| 6. | I'm certain I can understand the most difficult material presented in the readings for this course. | I'm certain I can understand the most difficult material presented in the readings for this clinical posting. | I am certain I can understand the most difficult content presented in the reading material for this clinical posting. |
| 7. | Getting a good grade in this class is the most satisfying thing for me right now. | Getting a good grade in this clinical posting is the most satisfying thing for me right now. | |
| 8. | When I take a test I think about items on other parts of the test I can't answer. | When I take an assessment in this clinical posting, I think about other parts of the assessment I can't answer or perform. | When I face an assessment in this clinical posting, I think about parts of the assessment I can't answer or perform well in. |
| 9. | It is my own fault if I don't learn the material in this course. | It is my own fault if I don't learn the study material in this clinical posting. | |
| 10. | It is important for me to learn the course material in this class. | It is important for me to learn the study material this clinical posting. | |
| 11. | The most important thing for me right now is improving my overall grade point average, so my main concern in this class is getting a good grade. | The most important thing for me right now is improve performance, so my main concern in this clinical posting is to ensure I pass this posting. | The most important thing for me right now is improve performance, so my main concern in this clinical posting is to ensure satisfactory performance. |
| 12. | I'm confident I can understand the basic concepts taught in this course. | I am confident I can learn the basic concepts taught in this clinical posting. | |
| 13. | If I can, I want to get better grades in this class than most of the other students. | If I can, I want to get better grades in this clinical posting than most of the other students. | |
| 14. | When I take a test I think of the consequences of failing. | When I take an assessment in this clinical posting, I think of the consequences of failing. | |
| 15. | I'm confident I can understand the most complex material presented by the instructor in this course. | I'm confident that I can understand the most complex material | |

| | | | |
|---|---|---|---|
| | | presented by the tutor in this clinical posting. | |
| 16. | In a class like this, I prefer course content that arouses my curiosity, even if it is difficult to learn. | In a class like this, I prefer content that arouses my curiosity, even if it is difficult to learn. | In a clinical posting like this, I prefer content that arouses my curiosity, even if it is difficult to learn. |
| 17. | I am very interested in the content area of this course. | I am very interested in the content area of this clinical posting. | |
| 18. | If I try hard enough, then I will understand the course material. | If I try hard enough, then I will understand the content in this clinical posting. | |
| 19. | I have an uneasy, upset feeling when I take a test. | I have an uneasy, upset feeling when I take an assessment in this clinical posting. | I have an uneasy, upset feeling when I face an assessment in this clinical posting. |
| 20. | I'm confident I can do an excellent job on the assignments and tests in this course. | I'm confident I can do an excellent job on the assessments in this clinical posting. | I am confident I can do an excellent job on the assessments in this clinical posting. |
| 21. | I expect to do well in this class. | I expect to do well in this clinical posting. | |
| 22. | The most satisfying thing for me in this course is trying to understand the content as thoroughly as possible. | The most satisfying thing for me in this clinical posting is trying to understand the content as thoroughly as possible. | |
| 23. | I think the course material in this class is useful for me to learn. | I think the content in this clinical posting is useful for me to learn. | |
| 24. | When I have the opportunity in this class, I choose course assignments that I can learn from even if they don't guarantee a good grade. | When I have the opportunity in this clinical posting, I choose tasks that I can learn from even if they don't guarantee a good grade. | When I have the opportunity in this clinical posting, I choose tasks that I can learn from even if they do not guarantee a good grade. |
| 25. | If I don't understand the course material, it is because I didn't try hard enough. | If I don't understand the clinical posting content, it is because I didn't try hard enough. | If I do not understand the clinical posting content, it is because I did not try hard enough. |
| 26. | I like the subject matter of this course. | I like the subject matter of this clinical posting. | |
| 27. | Understanding the subject matter of this course is very important to me. | Understanding the subject matter of this clinical posting is very important to me. | |
| 28. | I feel my heart beating fast when I take an exam. | I feel my heart beating fast when I take an assessment in this clinical posting. | I feel my heart beating fast when I face an assessment in this clinical posting. |
| 29. | I'm certain I can master the skills being taught in this class. | I am certain I can master the skills being taught in this clinical posting. | |
| 30. | I want to do well in this class because it is important to show my ability to my family, friends, employer, or others. | I want to do well in this clinical posting because it is important to show my ability to my family, friends, employer, or others. | |
| 31. | Considering the difficulty of this course, the teacher, and my skills, I think I will do well in this class. | Considering the difficulty of this clinical posting, the tutors, and my skills, I think I will do well. | |
| 32. | When I study the readings for this course, I outline the material to help me organize my thoughts. | When I study the material for this clinical posting, I outline the material to help me organize my thoughts. | |

| | | | |
|---|---|---|---|
| 33. | During class time I often miss important points because I'm thinking of other things. ® | During clinical posting time I often miss important points because I'm thinking of other things. | During this clinical posting time I often miss important points because I am thinking of other things. |
| 34. | When studying for this course, I often try to explain the material to a classmate or a friend. | When studying for this clinical posting, I often try to explain the material to a classmate or friend. | |
| 35. | I usually study in a place where I can concentrate on my course work. | I usually study in a place where I can concentrate on my work. | |
| 36. | When reading for this course, I make up questions to help focus my reading. | When reading for this clinical posting, I make up own questions to help focus my reading. | |
| 37. | I often feel so lazy or bored when I study for this class that I quit before I finish what I planned to do. ® | I often feel so lazy or bored when I study for this clinical posting that I quit before I finish what I planned to do. | |
| 38. | I often find myself questioning things I hear or read in this course to decide if I find them convincing. | I often find myself questioning things I hear or read in this clinical posting to decide if I find them convincing. | |
| 39. | When I study for this class, I practice saying the material to myself over and over. | When I study for this clinical posting, I practice saying the material to myself over and over. | |
| 40. | Even if I have trouble learning the material in this class, I try to do the work on my own, without help from anyone. ® | Even if I have trouble learning the content in this clinical posting, I try to do the work on my own, without help from anyone. | |
| 41. | When I become confused about something I'm reading for this class, I go back and try to figure it out. | When I become confused about something I'm reading for this clinical posting, I go back and try to figure it out. | When I become confused about something I am reading for this clinical posting, I go back and try to figure it out. |
| 42. | When I study for this course, I go through the readings and my class notes and try to find the most important ideas. | When I study for this clinical posting, I go through the readings and my notes and try to find the most important ideas. | |
| 43. | I make good use of my study time for this course. | I make good use of my study time for this clinical posting. | |
| 44. | If course materials are difficult to understand, I change the way I read the material. | If study materials are difficult to understand in this clinical posting, I change the way I read the material. | |
| 45. | I try to work with other students from this class to complete the course assignments. | I try to work with other students from this clinical posting to complete the clinical posting tasks. | |
| 46. | When studying for this class, I read my class notes and the course readings over and over again. | When studying for this clinical posting, I read my notes and the study material over and over again. | When studying for this clinical posting I do deliberate practice of my tasks repeatedly. |
| 47. | When a theory, interpretation, or conclusion is presented in class or in the readings, I try to decide if there is good supporting evidence. | When a theory, interpretation, or conclusion is presented in clinical posting or in the study material, I try to decide if there is good supporting evidence. | When a theory, interpretation, or conclusion is presented in this clinical posting or in the study material, I try to decide if there is good supporting evidence. |

| | | | |
|---|---|---|---|
| 48. | I work hard to do well in this class even if I don't like what we are doing. | I work hard to do well in this clinical posting even if I don’t like what we are doing. | I work hard to do well in this clinical posting even if I do not like what we are doing. |
| 49. | I make simple charts, diagrams, or tables to help me organize course material. | I make simple charts, diagrams, or tables to help me organize study material in this clinical posting. | |
| 50. | When studying for this course, I often set aside time to discuss the course material with a group of students from the class. | When studying for this clinical posting, I often set aside time to discuss study material with the group of students from the clinical posting. | |
| 51. | I treat the course material as a starting point and try to develop my own ideas about it. | I treat the study material in this clinical posting as a starting point and try to develop my own ideas about it. | |
| 52. | I find it hard to stick to a study schedule. | I find it hard to stick to a study schedule during clinical posting. | I find it hard to stick to a study schedule during this clinical posting. |
| 53. | When I study for this class, I pull together information from different sources, such as lectures, readings, and discussions. | When I study for this clinical posting, I pull together information from different sources, such as lectures, study materials and discussion. | |
| 54. | Before I study new course material thoroughly, I often skim it to see how it is organized. | Before I study new material thoroughly in this clinical posting, I often skim it to see how it is organized. | |
| 55. | I ask myself questions to make sure I understand the material I have been studying in this class. | I ask myself questions to make sure I understand the material I have been studying in this clinical posting. | |
| 56. | I try to change the way I study in order to fit the course requirements and instructor's teaching style. | I try to change the way I study in order to fit the clinical posting requirements and the tutor’s teaching style. | |
| 57. | I often find that I have been reading for class but don't know what it was all about. ® | I often find that I have been reading for this clinical posting but don't know what it was all about. | I often find that I have been reading for this clinical posting but do not know what it was all about. |
| 58. | I ask the instructor to clarify concepts I don't understand well. | I ask the tutor to clarify concepts I don’t understand well in this clinical posting. | I ask the tutor to clarify concepts I do not understand well in this clinical posting. |
| 59. | I memorize key words to remind me of important concepts in this class. | I memorize key words to remind me of important concepts in this clinical posting. | |
| 60. | When course work is difficult, I give up or only study the easy parts. ® | When clinical posting work is difficult, I either give up or only study the easy parts. | |
| 61. | I try to think through a topic and decide what I am supposed to learn from it rather than just reading it over when studying. | I try to think through a topic and decide what I am supposed to learn from it rather than just reading it over when studying for this clinical posting. | |
| 62. | I try to relate ideas in this subject to those in other courses whenever possible. | I try to relate ideas in this clinical posting to those in other clinical posting whenever possible. | |

| | | | |
|---|---|---|---|
| 63. | When I study for this course, I go over my class notes and make an outline of important concepts. | When I study for this clinical posting , I go over my notes and make an outline of important concepts. | |
| 64. | When reading for this class, I try to relate the material to what I already know. | When reading for this clinical posting , I try to relate the content to what I already know. | |
| 65. | I have a regular place set aside for studying. | I usually study in a place where I can concentrate on my work. | I have a regular place set aside for studying during this clinical posting. |
| 66. | I try to play around with ideas of my own related to what I am learning in this course. | I try to play around with ideas of my own related to what I am learning in this clinical posting. | |
| 67. | When I study for this course, I write brief summaries of the main ideas from the readings and the concepts from the lectures. | When I study for this clinical posting , I write brief summaries of the main ideas from the study materials and my notes. | |
| 68. | When I can't understand the material in this course, I ask another student in this class for help. | When I can't understand the material in this clinical posting, I ask another student in this posting for help. | When I cannot understand the material in this clinical posting, I ask another student in this posting for help. |
| 69. | I try to understand the material in this class by making connections between the readings and the concepts from the lectures. | I try to understand the material in this clinical posting by making connections between the study material and the concepts from the lectures. | |
| 70. | I make sure I keep up with the weekly readings and assignments for this course. | I make sure that I keep up with the weekly study material and tasks for this clinical posting. | |
| 71. | Whenever I read or hear an assertion or conclusion in this class, I think about possible alternatives. | Whenever I read or hear an assertion or conclusion in this clinical posting, I think about possible alternatives. | |
| 72. | I make lists of important terms for this course and memorize the lists. | I make lists of important items for this clinical posting and memorize the lists. | I make lists of important terms for this clinical posting and memorize the lists. |
| 73. | I attend class regularly. | I attend this clinical posting regularly. | |
| 74. | Even when course materials are dull and uninteresting, I manage to keep working until I finish. | Even when study materials are dull and uninteresting, I manage to keep working until I finish. | |
| 75. | I try to identify students in this class whom I can ask for help if necessary. | I try to identify students in this clinical posting whom I can ask for help if necessary. | |
| 76. | When studying for this course I try to determine which concepts I don't understand well. | When studying for this clinical posting I try to determine which concepts I don't understand well. | |
| 77. | I often find that I don't spend very much time on this course because of other activities. ® | I often find that I don't spend very much time on this clinical posting because of other activities. | I often find that I do not spend very much time on this clinical posting because of other activities. |
| 78. | When I study for this class, I set goals for myself in order to direct my activities in each study period. | When I study for this clinical posting, I set goals for myself in order to direct my activities in each study period. | |

| | | | |
|---|---|---|---|
| 79. | If I get confused taking notes in class, I make sure I sort it out afterwards. | If I get confused taking notes in this clinical posting, I make sure I sort it out afterwards. | |
| 80. | I rarely find time to review my notes or readings before an exam. ® | I rarely find time to review my notes or study material in this clinical posting before an assessment. | |
| 81. | I try to apply ideas from course readings in other class activities such as lecture and discussion. | I try to apply ideas from study materials in other class activities such as lecture and discussion in this clinical posting. | I try to apply ideas from study materials in other activities such as lecture and discussion in this clinical posting. |

**® :** reversed coded item
