# Supplementary material for: Validation of the Motivated Strategies for Learning Questionnaire among clinical clerkship students in Malaysia
Source: PLoS One. 2025 Apr 8;20(4):e0319763. doi: 10.1371/journal.pone.0319763 (PMC11978056; doi:10.1371/journal.pone.0319763)
Supplement: S1 Text — (PDF) [file pone.0319763.s002.pdf]

Amos output text with default settings and parameter estimates

CFA MOTIVATION.amw

Analysis Summary

Title

CFA motivation

Groups

Group number 1 (Group number 1)

Notes for Group (Group number 1)

The model is recursive.
Sample size = 349

Variable Summary (Group number 1)

Your model contains the following variables (Group number 1)

Observed, endogenous variables

Q1
Q16
Q22
Q24
Q7
Q11
Q13
Q30
Q4
Q10
Q17
Q23
Q26
Q27
Q2
Q9
Q18

Q25
Q5
Q6
Q12
Q15
Q20
Q21
Q29
Q31
Q3
Q8
Q14
Q19
Q28

Unobserved, exogenous variables

M_IGO
e1
e2
e3
e4
M_EGO
e5
e6
e7
e8
M_TV
e9
e10
e11
e12
e13
e14
M_CLB
e15
e16
e17
e18
M_SE
e19
e20
e21
e22
e23
e24
e25
e26
M_TA
e27
e28

e29
e30
e31

Variable counts (Group number 1)

**Number of variables in your model:**	68
**Number of observed variables:**	31
**Number of unobserved variables:**	37
**Number of exogenous variables:**	37
**Number of endogenous variables:**	31

Parameter Summary (Group number 1)

	Weights	Covariances	Variances	Means	Intercepts	Total
**Fixed**	37	0	0	0	0	37
**Labeled**	0	0	0	0	0	0
**Unlabeled**	25	15	37	0	0	77
**Total**	62	15	37	0	0	114

Models (Default model)

Computation of degrees of freedom (Default model)

**Number of distinct sample moments:**	496
**Number of distinct parameters to be estimated:**	77
**Degrees of freedom (496 - 77):**	419

Result (Default model)

Minimum was achieved
Chi-square = 1315.831
Degrees of freedom = 419
Probability level = .000

Scalar Estimates (Group number 1 - Default model)

Maximum Likelihood Estimates

Standardized Regression Weights: (Group number 1 - Default model)

			Estimate
Q1	<---	M_IGO	.587
Q16	<---	M_IGO	.560
Q22	<---	M_IGO	.770
Q24	<---	M_IGO	.714
Q7	<---	M_EGO	.547
Q11	<---	M_EGO	.607
Q13	<---	M_EGO	.600
Q30	<---	M_EGO	.448
Q4	<---	M_TV	.643
Q10	<---	M_TV	.591
Q17	<---	M_TV	.681
Q23	<---	M_TV	.773
Q26	<---	M_TV	.669
Q27	<---	M_TV	.794
Q2	<---	M_CLB	.669
Q9	<---	M_CLB	.416
Q18	<---	M_CLB	.758
Q25	<---	M_CLB	.381
Q5	<---	M_SE	.705
Q6	<---	M_SE	.630
Q12	<---	M_SE	.669
Q15	<---	M_SE	.695
Q20	<---	M_SE	.819
Q21	<---	M_SE	.807
Q29	<---	M_SE	.769
Q31	<---	M_SE	.753
Q3	<---	M_TA	.642
Q8	<---	M_TA	.647
Q14	<---	M_TA	.654
Q19	<---	M_TA	.691
Q28	<---	M_TA	.660

Correlations: (Group number 1 - Default model)

			Estimate
M_IGO	<-->	M_EGO	.739
M_IGO	<-->	M_TV	.882
M_IGO	<-->	M_CLB	.773
M_IGO	<-->	M_SE	.731
M_IGO	<-->	M_TA	.037
M_EGO	<-->	M_TV	.764
M_EGO	<-->	M_CLB	.761
M_EGO	<-->	M_SE	.567
M_EGO	<-->	M_TA	.303
M_TV	<-->	M_CLB	.917
M_TV	<-->	M_SE	.671
M_TV	<-->	M_TA	.106
M_CLB	<-->	M_SE	.547
M_CLB	<-->	M_TA	.247
M_SE	<-->	M_TA	-.307

Model Fit Summary

CMIN

Model	NPAR	CMIN	DF	P	CMIN/DF
Default model	77	1315.831	419	.000	3.140
Saturated model	496	.000	0		
Independence model	31	5496.376	465	.000	11.820

RMR, GFI

Model	RMR	GFI	AGFI	PGFI
Default model	.142	.786	.747	.664
Saturated model	.000	1.000		
Independence model	.458	.250	.200	.235

Baseline Comparisons

Model	NFI Delta1	RFI rho1	IFI Delta2	TLI rho2	CFI
Default model	.761	.734	.823	.802	.822
Saturated model	1.000		1.000		1.000
Independence model	.000	.000	.000	.000	.000

RMSEA

Model	RMSEA	LO 90	HI 90	PCLOSE
Default model	.078	.074	.083	.000
Independence model	.176	.172	.181	.000

Execution time summary

Minimization: .023
Miscellaneous: .541
Bootstrap: .000
Total: .564

CFA MOTIVATION (Re-Specified)

Analysis Summary

Title

CFA Motivation re-specified

Groups

Group number 1 (Group number 1)

Notes for Group (Group number 1)

The model is recursive.
Sample size = 349

Variable Summary (Group number 1)

Your model contains the following variables (Group number 1)

Observed, endogenous variables
Q1
Q16
Q22
Q24
Q7
Q11
Q13
Q30
Q4
Q10
Q17
Q23
Q26
Q27
Q2
Q9
Q18
Q5
Q6
Q12
Q15
Q20

Q21
Q29
Q31
Q3
Q8
Q14
Q19
Q28
Q25
Unobserved, exogenous variables
M_IGO
e1
e2
e3
e4
M_EGO
e5
e6
e7
e8
M_TV
e9
e10
e11
e12
e13
e14
M_CLB
e15
e16
e17
M_SE
e19
e20
e21
e22
e23
e24
e25
e26
M_TA
e27
e28
e29
e30
e31
e18

Variable counts (Group number 1)

**Number of variables in your model:**	68
**Number of observed variables:**	31
**Number of unobserved variables:**	37
**Number of exogenous variables:**	37
**Number of endogenous variables:**	31

Parameter Summary (Group number 1)

	Weights	Covariances	Variances	Means	Intercepts	Total
**Fixed**	37	0	0	0	0	37
**Labeled**	0	0	0	0	0	0
**Unlabeled**	25	16	37	0	0	78
**Total**	62	16	37	0	0	115

Models (Default model)

Computation of degrees of freedom (Default model)

**Number of distinct sample moments:**	496
**Number of distinct parameters to be estimated:**	78
**Degrees of freedom (496 - 78):**	418

Result (Default model)

Minimum was achieved
Chi-square = 1231.576
Degrees of freedom = 418
Probability level = .000

Scalar Estimates (Group number 1 - Default model)

Maximum Likelihood Estimates

Standardized Regression Weights:

			Estimate
Q1	<---	M_IGO	.587
Q16	<---	M_IGO	.554
Q22	<---	M_IGO	.775
Q24	<---	M_IGO	.711
Q7	<---	M_EGO	.542
Q11	<---	M_EGO	.615
Q13	<---	M_EGO	.593
Q30	<---	M_EGO	.445
Q4	<---	M_TV	.639
Q10	<---	M_TV	.608
Q17	<---	M_TV	.635
Q23	<---	M_TV	.786
Q26	<---	M_TV	.621
Q27	<---	M_TV	.800
Q2	<---	M_CLB	.673
Q9	<---	M_CLB	.416
Q18	<---	M_CLB	.753
Q5	<---	M_SE	.706
Q6	<---	M_SE	.631
Q12	<---	M_SE	.668
Q15	<---	M_SE	.696
Q20	<---	M_SE	.821
Q21	<---	M_SE	.806
Q29	<---	M_SE	.767
Q31	<---	M_SE	.753
Q3	<---	M_TA	.642
Q8	<---	M_TA	.648
Q14	<---	M_TA	.654
Q19	<---	M_TA	.691
Q28	<---	M_TA	.660
Q25	<---	M_CLB	.382

Correlations:

			Estimate
M_IGO	<-->	M_EGO	.743
M_IGO	<-->	M_TV	.880
M_IGO	<-->	M_CLB	.775
M_IGO	<-->	M_SE	.730
M_IGO	<-->	M_TA	.038
M_EGO	<-->	M_TV	.781
M_EGO	<-->	M_CLB	.765
M_EGO	<-->	M_SE	.566
M_EGO	<-->	M_TA	.306
M_TV	<-->	M_CLB	.943
M_TV	<-->	M_SE	.642
M_TV	<-->	M_TA	.126
M_CLB	<-->	M_SE	.546
M_CLB	<-->	M_TA	.250
M_SE	<-->	M_TA	-.307
e11	<-->	e13	.490

Model Fit Summary

CMIN

Model	NPAR	CMIN	DF	P	CMIN/DF
**Default model**	78	1231.576	418	.000	2.946
**Saturated model**	496	.000	0		
**Independence model**	31	5496.376	465	.000	11.820

RMR, GFI

Model	RMR	GFI	AGFI	PGFI
**Default model**	.143	.800	.762	.674
**Saturated model**	.000	1.000		
**Independence model**	.458	.250	.200	.235

Baseline Comparisons

Model	NFI Delta1	RFI rho1	IFI Delta2	TLI rho2	CFI
**Default model**	.776	.751	.840	.820	.838
**Saturated model**	1.000		1.000		1.000
**Independence model**	.000	.000	.000	.000	.000

RMSEA

Model	RMSEA	LO 90	HI 90	PCLOSE
Default model	.075	.070	.080	.000
Independence model	.176	.172	.181	.000

Execution time summary

Minimization: .039
Miscellaneous: .663
Bootstrap: .000
Total: .702

Syntax for MOT (after re-specification)

Q1 = () e1 + (.59) M_IGO
Q10 = () e10 + (.61) M_TV
Q11 = (.62) M_EGO + () e6
Q12 = (.67) M_SE + () e21
Q13 = (.59) M_EGO + () e7
Q14 = () e29 + (.65) M_TA
Q15 = (.70) M_SE + () e22
Q16 = (.55) M_IGO + () e2
Q17 = (.64) M_TV + () e11
Q18 = () e17 + (.75) M_CLB
Q19 = (.69) M_TA + () e30
Q2 = (.67) M_CLB + () e15
Q20 = () e23 + (.82) M_SE
Q21 = (.81) M_SE + () e24
Q22 = () e3 + (.78) M_IGO
Q23 = (.79) M_TV + () e12
Q24 = () e4 + (.71) M_IGO
Q25 = () e18 + (.38) M_CLB
Q26 = (.62) M_TV + () e13
Q27 = () e14 + (.80) M_TV
Q28 = () e31 + (.66) M_TA
Q29 = () e25 + (.77) M_SE
Q3 = () e27 + (.64) M_TA
Q30 = (.44) M_EGO + () e8
Q31 = () e26 + (.75) M_SE
Q4 = (.64) M_TV + () e9
Q5 = () e19 + (.71) M_SE
Q6 = () e20 + (.63) M_SE
Q7 = () e5 + (.54) M_EGO
Q8 = () e28 + (.65) M_TA
Q9 = () e16 + (.42) M_CLB

M_IGO <> M_EGO (.74)
M_IGO <> M_TV (.88)
M_IGO <> M_CLB (.78)
M_IGO <> M_SE (.73)
M_IGO <> M_TA (.04)
M_EGO <> M_TV (.78)
M_EGO <> M_CLB (.77)
M_EGO <> M_SE (.57)
M_EGO <> M_TA (.31)
M_TV <> M_CLB (.94)
M_TV <> M_SE (.64)
M_TV <> M_TA (.13)
M_CLB <> M_SE (.55)
M_CLB <> M_TA (.25)
M_SE <> M_TA (-.31)
e11 <> e13 (.49)

CFA LS.amw

Analysis Summary

Title

CFA LS

Groups

Group number 1 (Group number 1)

Notes for Group (Group number 1)

The model is recursive.
Sample size = 349

Variable Summary (Group number 1)

Your model contains the following variables (Group number 1)

Observed, endogenous variables
Q39
Q46
Q59
Q72
Q53
Q62
Q64
Q67
Q69
Q81
Q63
Q49
Q42
Q32
Q71
Q66
Q51
Q47
Q38
Q40r
Q58
Q68

Q75
Q34
Q45
Q50
Q33r
Q36
Q41
Q44
Q54
Q55
Q56
Q57r
Q61
Q76
Q78
Q79
Q35
Q43
Q52r
Q65
Q70
Q73
Q77r
Q80r
Q74
Q60r
Q48
Q37r
Unobserved, exogenous variables
LS_Reh
e1
e2
e3
e4
LS_Elab
e5
e6
e7
e8
e9
e10
LS_Org
e11
e12
e13
e14
LS_CrTh
e15
e16
e17

e18
e19
LS_HS
e20
e21
e22
e23
LS_PL
e24
e25
e26
LS_Met_SR
e27
e28
e29
e30
e31
e32
e33
e34
e35
e36
e37
e38
LS_T_StEnv
e39
e40
e41
e42
e43
e44
e45
e46
LS_Ef_Reg
e47
e48
e49
e50

Variable counts (Group number 1)

**Number of variables in your model:**	109
**Number of observed variables:**	50
**Number of unobserved variables:**	59
**Number of exogenous variables:**	59
**Number of endogenous variables:**	50

Parameter Summary (Group number 1)

	Weights	Covariances	Variances	Means	Intercepts	Total
**Fixed**	59	0	0	0	0	59
**Labeled**	0	0	0	0	0	0
**Unlabeled**	41	36	59	0	0	136
**Total**	100	36	59	0	0	195

Models

Computation of degrees of freedom (Default model)

**Number of distinct sample moments:**	1275
**Number of distinct parameters to be estimated:**	136
**Degrees of freedom (1275 - 136):**	1139

Result (Default model)

Minimum was achieved
Chi-square = 3402.215
Degrees of freedom = 1139
Probability level = .000

Scalar Estimates (Group number 1 - Default model)

Maximum Likelihood Estimates

Standardized Regression Weights: (Group number 1 - Default model)

			Estimate
Q39	<---	LS_Reh	.480
Q46	<---	LS_Reh	.682
Q59	<---	LS_Reh	.630
Q72	<---	LS_Reh	.522
Q53	<---	LS_Elab	.624
Q62	<---	LS_Elab	.677
Q64	<---	LS_Elab	.730
Q67	<---	LS_Elab	.582
Q69	<---	LS_Elab	.804
Q81	<---	LS_Elab	.710
Q63	<---	LS_Org	.669
Q49	<---	LS_Org	.445

			Estimate
Q42	<---	LS_Org	.730
Q32	<---	LS_Org	.621
Q71	<---	LS_CrTh	.744
Q66	<---	LS_CrTh	.704
Q51	<---	LS_CrTh	.681
Q47	<---	LS_CrTh	.596
Q38	<---	LS_CrTh	.520
Q40r	<---	LS_HS	.104
Q58	<---	LS_HS	.492
Q68	<---	LS_HS	.821
Q75	<---	LS_HS	.741
Q34	<---	LS_PL	.537
Q45	<---	LS_PL	.693
Q50	<---	LS_PL	.548
Q33r	<---	LS_Met_SR	.171
Q36	<---	LS_Met_SR	.517
Q41	<---	LS_Met_SR	.606
Q44	<---	LS_Met_SR	.652
Q54	<---	LS_Met_SR	.556
Q55	<---	LS_Met_SR	.674
Q56	<---	LS_Met_SR	.611
Q57r	<---	LS_Met_SR	.065
Q61	<---	LS_Met_SR	.559
Q76	<---	LS_Met_SR	.740
Q78	<---	LS_Met_SR	.743
Q79	<---	LS_Met_SR	.674
Q35	<---	LS_T_StEnv	.495
Q43	<---	LS_T_StEnv	.633
Q52r	<---	LS_T_StEnv	.219
Q65	<---	LS_T_StEnv	.434
Q70	<---	LS_T_StEnv	.589
Q73	<---	LS_T_StEnv	.533
Q77r	<---	LS_T_StEnv	.295
Q80r	<---	LS_T_StEnv	.220
Q74	<---	LS_Ef_Reg	.650
Q60r	<---	LS_Ef_Reg	.476
Q48	<---	LS_Ef_Reg	.381
Q37r	<---	LS_Ef_Reg	.580

Correlations: (Group number 1 - Default model)

			Estimate
LS_Reh	<-->	LS_Elab	.852
LS_Reh	<-->	LS_Org	.906
LS_Reh	<-->	LS_CrTh	.816
LS_Reh	<-->	LS_Ef_Reg	.683
LS_Reh	<-->	LS_HS	.634
LS_Reh	<-->	LS_PL	.930
LS_Reh	<-->	LS_Met_SR	.954
LS_Reh	<-->	LS_T_StEnv	.809
LS_Elab	<-->	LS_Org	.891
LS_Elab	<-->	LS_CrTh	.727
LS_Elab	<-->	LS_Ef_Reg	.693
LS_Elab	<-->	LS_HS	.669
LS_Elab	<-->	LS_PL	.784
LS_Elab	<-->	LS_Met_SR	.922
LS_Elab	<-->	LS_T_StEnv	.845
LS_Org	<-->	LS_CrTh	.679
LS_Org	<-->	LS_Ef_Reg	.697
LS_Org	<-->	LS_HS	.465
LS_Org	<-->	LS_PL	.718
LS_Org	<-->	LS_Met_SR	.838
LS_Org	<-->	LS_T_StEnv	.789
LS_CrTh	<-->	LS_Ef_Reg	.466
LS_CrTh	<-->	LS_HS	.515
LS_CrTh	<-->	LS_PL	.767
LS_CrTh	<-->	LS_Met_SR	.867
LS_CrTh	<-->	LS_T_StEnv	.650
LS_HS	<-->	LS_Ef_Reg	.420
LS_PL	<-->	LS_Ef_Reg	.600
LS_Met_SR	<-->	LS_Ef_Reg	.730
LS_T_StEnv	<-->	LS_Ef_Reg	1.061
LS_HS	<-->	LS_PL	.869
LS_HS	<-->	LS_Met_SR	.628
LS_HS	<-->	LS_T_StEnv	.567
LS_PL	<-->	LS_Met_SR	.824
LS_PL	<-->	LS_T_StEnv	.647
LS_Met_SR	<-->	LS_T_StEnv	.909

Model Fit Summary

CMIN

Model	NPAR	CMIN	DF	P	CMIN/DF
**Default model**	136	3402.215	1139	.000	2.987
**Saturated model**	1275	.000	0		
**Independence model**	50	9030.670	1225	.000	7.372

RMR, GFI

Model	RMR	GFI	AGFI	PGFI
**Default model**	.192	.675	.637	.603
**Saturated model**	.000	1.000		
**Independence model**	.518	.186	.153	.179

Baseline Comparisons

Model	NFI Delta1	RFI rho1	IFI Delta2	TLI rho2	CFI
**Default model**	.623	.595	.713	.688	.710
**Saturated model**	1.000		1.000		1.000
**Independence model**	.000	.000	.000	.000	.000

RMSEA

Model	RMSEA	LO 90	HI 90	PCLOSE
**Default model**	.076	.073	.078	.000
**Independence model**	.135	.133	.138	.000

Execution time summary

Minimization: .093
Miscellaneous: .517
Bootstrap: .000
Total: .610

CFA LS re-specified.amw

Analysis Summary

Title

CFA LS

Groups

Group number 1 (Group number 1)

Notes for Group (Group number 1)

The model is recursive.
Sample size = 349

Variable Summary (Group number 1)

Your model contains the following variables (Group number 1)

Observed, endogenous variables
Q39
Q46
Q59
Q72
Q53
Q62
Q64
Q67
Q69
Q81
Q63
Q49
Q42
Q32
Q71
Q66
Q51
Q47
Q38
Q58
Q68
Q75

Q34
Q45
Q50
Q36
Q41
Q44
Q54
Q55
Q56
Q61
Q76
Q78
Q79
Q35
Q43
Q65
Q70
Q73
Q74
Q60r
Q48
Q37r
Unobserved, exogenous variables
LS_Reh
e1
e2
e3
e4
LS_Elab
e5
e6
e7
e8
e9
e10
LS_Org
e11
e12
e13
e14
LS_CrTh
e15
e16
e17
e18
e19
LS_HS
e21
e22
e23

LS_PL
e24
e25
e26
LS_Met_SR
e28
e29
e30
e31
e32
e33
e35
e36
e37
e38
LS_T_StEnv
e39
e40
e42
e43
e44
LS_Ef_Reg
e47
e48
e49
e50

Variable counts (Group number 1)

**Number of variables in your model:**	97
**Number of observed variables:**	44
**Number of unobserved variables:**	53
**Number of exogenous variables:**	53
**Number of endogenous variables:**	44

Parameter Summary (Group number 1)

	Weights	Covariances	Variances	Means	Intercepts	Total
**Fixed**	53	0	0	0	0	53
**Labeled**	0	0	0	0	0	0
**Unlabeled**	35	37	53	0	0	125
**Total**	88	37	53	0	0	178

Models

Default model (Default model)

Computation of degrees of freedom (Default model)

**Number of distinct sample moments:**	990
**Number of distinct parameters to be estimated:**	125
**Degrees of freedom (990 - 125):**	865

Result (Default model)

Minimum was achieved
Chi-square = 2421.568
Degrees of freedom = 865
Probability level = .000

Scalar Estimates (Group number 1 - Default model)

Maximum Likelihood Estimates

Standardized Regression Weights:

			Estimate
Q39	<---	LS_Reh	.476
Q46	<---	LS_Reh	.682
Q59	<---	LS_Reh	.630
Q72	<---	LS_Reh	.525
Q53	<---	LS_Elab	.621
Q62	<---	LS_Elab	.647
Q64	<---	LS_Elab	.703
Q67	<---	LS_Elab	.598
Q69	<---	LS_Elab	.798
Q81	<---	LS_Elab	.708
Q63	<---	LS_Org	.669
Q49	<---	LS_Org	.447
Q42	<---	LS_Org	.730
Q32	<---	LS_Org	.621
Q71	<---	LS_CrTh	.745
Q66	<---	LS_CrTh	.704
Q51	<---	LS_CrTh	.683
Q47	<---	LS_CrTh	.594
Q38	<---	LS_CrTh	.519
Q58	<---	LS_HS	.502

			Estimate
Q68	<---	LS_HS	.816
Q75	<---	LS_HS	.737
Q34	<---	LS_PL	.538
Q45	<---	LS_PL	.690
Q50	<---	LS_PL	.551
Q36	<---	LS_Met_SR	.521
Q41	<---	LS_Met_SR	.601
Q44	<---	LS_Met_SR	.653
Q54	<---	LS_Met_SR	.558
Q55	<---	LS_Met_SR	.677
Q56	<---	LS_Met_SR	.612
Q61	<---	LS_Met_SR	.560
Q76	<---	LS_Met_SR	.739
Q78	<---	LS_Met_SR	.744
Q79	<---	LS_Met_SR	.672
Q35	<---	LS_T_StEnv	.497
Q43	<---	LS_T_StEnv	.626
Q65	<---	LS_T_StEnv	.457
Q70	<---	LS_T_StEnv	.598
Q73	<---	LS_T_StEnv	.525
Q74	<---	LS_Ef_Reg	.683
Q60r	<---	LS_Ef_Reg	.432
Q48	<---	LS_Ef_Reg	.432
Q37r	<---	LS_Ef_Reg	.515

Correlations:

			Estimate
LS_Reh	<-->	LS_Elab	.862
LS_Reh	<-->	LS_Org	.906
LS_Reh	<-->	LS_CrTh	.816
LS_Reh	<-->	LS_Ef_Reg	.735
LS_Reh	<-->	LS_HS	.644
LS_Reh	<-->	LS_PL	.931
LS_Reh	<-->	LS_Met_SR	.956
LS_Reh	<-->	LS_T_StEnv	.891
LS_Elab	<-->	LS_Org	.904
LS_Elab	<-->	LS_CrTh	.735
LS_Elab	<-->	LS_Ef_Reg	.735
LS_Elab	<-->	LS_HS	.693
LS_Elab	<-->	LS_PL	.790
LS_Elab	<-->	LS_Met_SR	.934
LS_Elab	<-->	LS_T_StEnv	.895

			Estimate
LS_Org	<-->	LS_CrTh	.679
LS_Org	<-->	LS_Ef_Reg	.735
LS_Org	<-->	LS_HS	.474
LS_Org	<-->	LS_PL	.719
LS_Org	<-->	LS_Met_SR	.838
LS_Org	<-->	LS_T_StEnv	.816
LS_CrTh	<-->	LS_Ef_Reg	.510
LS_CrTh	<-->	LS_HS	.524
LS_CrTh	<-->	LS_PL	.770
LS_CrTh	<-->	LS_Met_SR	.872
LS_CrTh	<-->	LS_T_StEnv	.731
LS_HS	<-->	LS_Ef_Reg	.457
LS_PL	<-->	LS_Ef_Reg	.623
LS_Met_SR	<-->	LS_Ef_Reg	.749
LS_T_StEnv	<-->	LS_Ef_Reg	1.018
LS_HS	<-->	LS_PL	.872
LS_HS	<-->	LS_Met_SR	.638
LS_HS	<-->	LS_T_StEnv	.620
LS_PL	<-->	LS_Met_SR	.825
LS_PL	<-->	LS_T_StEnv	.699
LS_Met_SR	<-->	LS_T_StEnv	.940
e6	<-->	e7	.311

Model Fit Summary

CMIN

Model	NPAR	CMIN	DF	P	CMIN/DF
**Default model**	125	2421.568	865	.000	2.800
**Saturated model**	990	.000	0		
**Independence model**	44	8019.336	946	.000	8.477

RMR, GFI

Model	RMR	GFI	AGFI	PGFI
**Default model**	.139	.741	.703	.647
**Saturated model**	.000	1.000		
**Independence model**	.563	.174	.136	.166

Baseline Comparisons

Model	NFI Delta1	RFI rho1	IFI Delta2	TLI rho2	CFI
**Default model**	.698	.670	.782	.759	.780
**Saturated model**	1.000		1.000		1.000
**Independence model**	.000	.000	.000	.000	.000

RMSEA

Model	RMSEA	LO 90	HI 90	PCLOSE
**Default model**	.072	.069	.075	.000
**Independence model**	.147	.144	.150	.000

Execution time summary

**Minimization:**	.041
**Miscellaneous:**	.761
**Bootstrap:**	.000
**Total:**	.802

Syntax for LS (after re-specification)

Q32 = () e14 + (.62) LS_Org
Q34 = () e24 + (.54) LS_PL
Q35 = () e39 + (.50) LS_T_StEnv
Q36 = () e28 + (.52) LS_Met_SR
Q37r = () e50 + (.52) LS_Ef_Reg
Q38 = () e19 + (.52) LS_CrTh
Q39 = (.48) LS_Reh + () e1
Q41 = () e29 + (.60) LS_Met_SR
Q42 = (.73) LS_Org + () e13
Q43 = () e40 + (.63) LS_T_StEnv
Q44 = () e30 + (.65) LS_Met_SR
Q45 = () e25 + (.69) LS_PL
Q46 = () e2 + (.68) LS_Reh
Q47 = () e18 + (.59) LS_CrTh
Q48 = () e49 + (.43) LS_Ef_Reg
Q49 = () e12 + (.45) LS_Org
Q50 = (.55) LS_PL + () e26
Q51 = (.68) LS_CrTh + () e17
Q53 = (.62) LS_Elab + () e5
Q54 = (.56) LS_Met_SR + () e31
Q55 = () e32 + (.68) LS_Met_SR
Q56 = () e33 + (.61) LS_Met_SR
Q58 = (.50) LS_HS + () e21
Q59 = (.63) LS_Reh + () e3
Q60r = (.43) LS_Ef_Reg + () e48
Q61 = (.56) LS_Met_SR + () e35
Q62 = (.65) LS_Elab + () e6
Q63 = () e11 + (.67) LS_Org
Q64 = () e7 + (.70) LS_Elab
Q65 = () e42 + (.46) LS_T_StEnv
Q66 = () e16 + (.70) LS_CrTh
Q67 = (.60) LS_Elab + () e8
Q68 = () e22 + (.82) LS_HS
Q69 = (.80) LS_Elab + () e9
Q70 = () e43 + (.60) LS_T_StEnv
Q71 = () e15 + (.74) LS_CrTh
Q72 = (.53) LS_Reh + () e4
Q73 = (.52) LS_T_StEnv + () e44
Q74 = (.68) LS_Ef_Reg + () e47
Q75 = () e23 + (.74) LS_HS
Q76 = () e36 + (.74) LS_Met_SR
Q78 = () e37 + (.74) LS_Met_SR
Q79 = () e38 + (.67) LS_Met_SR
Q81 = (.71) LS_Elab + () e10

LS_Elab <> LS_Reh (.86)
LS_Org <> LS_Reh (.91)
LS_CrTh <> LS_Reh (.82)

LS_Ef_Reg <> LS_Reh (.74)
LS_Reh <> LS_HS (.64)
LS_Reh <> LS_PL (.93)
LS_Reh <> LS_Met_SR (.96)
LS_Reh <> LS_T_StEnv (.89)
LS_Org <> LS_Elab (.90)
LS_CrTh <> LS_Elab (.74)
LS_Ef_Reg <> LS_Elab (.74)
LS_Elab <> LS_HS (.69)
LS_Elab <> LS_PL (.79)
LS_Elab <> LS_Met_SR (.93)
LS_T_StEnv <> LS_Elab (.90)
LS_CrTh <> LS_Org (.68)
LS_Ef_Reg <> LS_Org (.74)
LS_Org <> LS_HS (.47)
LS_Org <> LS_PL (.72)
LS_Org <> LS_Met_SR (.84)
LS_T_StEnv <> LS_Org (.82)
LS_Ef_Reg <> LS_CrTh (.51)
LS_CrTh <> LS_HS (.52)
LS_CrTh <> LS_PL (.77)
LS_Met_SR <> LS_CrTh (.87)
LS_T_StEnv <> LS_CrTh (.73)
LS_Ef_Reg <> LS_HS (.46)
LS_PL <> LS_Ef_Reg (.62)
LS_Met_SR <> LS_Ef_Reg (.75)
LS_T_StEnv <> LS_Ef_Reg (1.02)
LS_HS <> LS_PL (.87)
LS_HS <> LS_Met_SR (.64)
LS_HS <> LS_T_StEnv (.62)
LS_PL <> LS_Met_SR (.83)
LS_PL <> LS_T_StEnv (.70)
LS_Met_SR <> LS_T_StEnv (.94)
e6 <> e7 (.31)
