# Supplementary material for: Validation of the Motivated Strategies for Learning Questionnaire among clinical clerkship students in Malaysia
Source: PLoS One. 2025 Apr 8;20(4):e0319763. doi: 10.1371/journal.pone.0319763 (PMC11978056; doi:10.1371/journal.pone.0319763)
Supplement: S2 Appendix — (PDF) [file pone.0319763.s004.pdf]

MSLQ-CL (Adapted and validated to be used in clinical clerkship context)

General Description

The 75-item (total of 15 sub-scales) modified MSLQ consists of two major sections: Motivation and Learning strategies. Motivation section contains 31 items (six sub-scales) that assess students' goals and value beliefs for a course, their beliefs about their skill to succeed in a course, and their anxiety about tests in a course (Pintrich & De Groot, 1990). The Learning strategies section contains 44 items (nine sub-scales) that includes items regarding students' use of different cognitive and metacognitive strategies and student management of different resources (Pintrich & De Groot, 1990). The items are rated on a 7-point Likert scale from 1 being "not at all true of me" to 7 being "very true of me". Items marked as "reversed" are reverse coded items for which the ratings have to be reversed before a Scale scores are constructed by taking the mean of the items that make up that scale or sub-scale.

MOTIVATION

(i) Intrinsic Goal Orientation

(1) In a clinical posting, I prefer study material that really challenges me so I can learn new things.
(16) In a class like this, I prefer content that arouses my curiosity, even if it is difficult to learn.
(22) The most satisfying thing for me in this clinical posting is trying to understand the content as thoroughly as possible.
(24) When I have the opportunity in this clinical posting, I choose tasks that I can learn from even if they don't guarantee a good grade.

(ii) Extrinsic Goal Orientation

(7) Getting a good grade in this clinical posting is the most satisfying thing for me right now.
(11) The most important thing for me right now is improve performance, so my main concern in this clinical posting is to ensure I pass this posting.
(13) If I can, I want to get better grades in this clinical posting than most of the other students.
(30) I want to do well in this clinical posting because it is important to show my ability to my family, friends, employer, or others.

(iii) Task Value

(4) I think I will be able to use what I learn in this clinical posting in other clinical posting.
(10) It is important for me to learn the study material in this clinical posting.
(17) I am very interested in the content area of this clinical posting.
(23) I think the content in this clinical posting is useful for me to learn.
(26) I like the subject matter of this clinical posting.
(27) Understanding the subject matter of this clinical posting is very important to me.

(iv) Control of Learning Beliefs

(2) If I study in appropriate ways, then I will be able to learn the content in this clinical posting.
(9) It is my own fault if I don't learn the study material in this clinical posting.
(18) If I try hard enough, then I will understand the content in this clinical posting.
(25) If I don't understand the clinical posting content, it is because I didn't try hard enough.

(v) Self-Efficacy

(5) I believe I will receive an excellent grade for this clinical posting.
(6) I'm certain I can understand the most difficult material presented in the readings for this clinical posting.
(12) I am confident I can learn the basic concepts taught in this clinical posting.
(15) I'm confident that I can understand the most complex material presented by the tutor in this clinical posting.
(20) I'm confident I can do an excellent job on the assessments in this clinical posting.
(21) I expect to do well in this clinical posting.
(29) I am certain I can master the skills being taught in this clinical posting.
(31) Considering the difficulty of this clinical posting, the tutors, and my skills, I think I will do well.

(vi) Test Anxiety

(3) When I take an assessment in this clinical posting, I think about how poorly I am doing compared to other students.
(8) When I take an assessment in this clinical posting, I think about other parts of the assessment I can't answer or perform.
(14) When I take an assessment in this clinical posting, I think of the consequences of failing.
(19) I have an uneasy, upset feeling when I take an assessment in this clinical posting
(28) I feel my heart beating fast when I take an assessment in this clinical posting.

LEARNING STRATEGIES

(i) Rehearsal

(38) When I study for this clinical posting, I practice saying the material to myself over and over.
(44) When studying for this clinical posting, I read my notes and the study material over and over again.
(55) I memorize key words to remind me of important concepts in this clinical posting.
(68) I make lists of important items for this clinical posting and memorize the lists.

(ii) Elaboration

(50) When I study for this clinical posting, I pull together information from different sources, such as lectures, study materials and discussion.
(58) I try to relate ideas in this clinical posting to those in other clinical posting whenever possible.
(60) When reading for this clinical posting , I try to relate the content to what I already know.
(63) When I study for this clinical posting , I write brief summaries of the main ideas from the study materials and my notes.
(65) I try to understand the material in this clinical posting by making connections between the study material and the concepts from the lectures.
(75) I try to apply ideas from study materials in other class activities such as lecture and discussion in this clinical posting.

(iii) Organisation

(32) When I study the material for this clinical posting, I outline the material to help me organize my thoughts.
(40) When I study for this clinical posting, I go through the readings and my notes and try to find the most important ideas.
(47) I make simple charts, diagrams, or tables to help me organize study material in this clinical posting.
(59) When I study for this clinical posting , I go over my notes and make an outline of important concepts.

(iv) Critical Thinking

(37) I often find myself questioning things I hear or read in this clinical posting to decide if I find them convincing.
(45) When a theory, interpretation, or conclusion is presented in clinical posting or in the study material, I try to decide if there is good supporting evidence.
(49) I treat the study material in this clinical posting as a starting point and try to develop my own ideas about it.

(62) When I study for this clinical posting , I write brief summaries of the main ideas from the study materials and my notes.
(67) Whenever I read or hear an assertion or conclusion in this clinical posting, I think about possible alternatives.

(v) Metacognitive Self-regulation

(35) When reading for this clinical posting, I make up own questions to help focus my reading.
(39) When I become confused about something I'm reading for this clinical posting, I go back and try to figure it out.
(42) If study materials are difficult to understand in this clinical posting, I change the way I read the material.
(51) Before I study new material thoroughly in this clinical posting, I often skim it to see how it is organized.
(52) I ask myself questions to make sure I understand the material I have been studying in this clinical posting.
(53) I try to change the way I study in order to fit the clinical posting requirements and the tutor's teaching style.
(57) I try to think through a topic and decide what I am supposed to learn from it rather than just reading it over when studying for this clinical posting.
(72) When studying for this clinical posting I try to determine which concepts I don't understand well.
(73) When I study for this clinical posting, I set goals for myself in order to direct my activities in each study period.
(74) If I get confused taking notes in this clinical posting, I make sure I sort it out afterwards.

(vi) Time and Study Environment

(34) I usually study in a place where I can concentrate on my work.
(41) I make good use of my study time for this clinical posting.
(61) I have a regular place set aside for studying.
(66) I make sure that I keep up with the weekly study material and tasks for this clinical posting.
(69) I attend this clinical posting regularly.

(vii) Effort Regulation

(36) I often feel so lazy or bored when I study for this clinical posting that I quit before I finish what I planned to do. (REVERSED)
(46) I work hard to do well in this clinical posting even if I don't like what we are doing.
(56) When clinical posting work is difficult, I either give up or only study the easy parts. (REVERSED)
(70) Even when study materials are dull and uninteresting, I manage to keep working until I finish.

(viii) Peer Learning

(33) When studying for this clinical posting, I often try to explain the material to a classmate or friend.
(43) I try to work with other students from this clinical posting to complete the clinical posting tasks.
(48) When studying for this clinical posting, I often set aside time to discuss study material with the group of students from the clinical posting.

(ix) Help Seeking

(54) I ask the tutor to clarify concepts I don't understand well in this clinical posting.
(64) When I can't understand the material in this clinical posting, I ask another student in this posting for help.
(71) I try to identify students in this clinical posting whom I can ask for help if necessary.
